# Association between exposure to Efavirenz and substrates of dysrhythmia in HIV‐infected young adults

**DOI:** 10.1002/clc.23705

**Published:** 2021-07-30

**Authors:** Zahra Hosseini, Reza Mollazadeh, Seyed‐Ali Dehghan‐Manshadi, Mehrnaz Mohebi, Masoud Eslami, Seyed‐Ali Sadre‐Bafghi, Ali Akbari, Saeed Ghodsi

**Affiliations:** ^1^ Department of Cardiology Imam Khomeini Hospital Complex, Tehran University of Medical Sciences Tehran Iran; ^2^ Department of Infectious and Tropical diseases HIV‐AIDS Research Center, Imam Khomeini Hospital Complex, Tehran University of Medical Sciences Tehran Iran; ^3^ Department of Research Tehran Heart Center, Tehran University of Medical Sciences Tehran Iran; ^4^ Biomedical Engineering Hill‐Rom (Mortara) Sale and Technical Expert Iranbehdasht Co Tehran Iran

**Keywords:** antiretroviral treatment, cardiovascular disease, dysrhythmia, ECG Holter monitoring, Efavirenz, HIV

## Abstract

**Background:**

Dysrhythmia and sudden cardiac arrest occur more likely in HIV patients than healthy subjects. Thus, we need to examine dysrhythmias adverse effects of medications including Efavirenz as early as possible especially in young subjects.

**Hypothesis:**

Efavirenz might have contributed to increased risk of developing common types of dysrhythmia in young HIV infected patients.

**Methods:**

We performed a retrospective cohort study among 62 patients on Efavirenz and 38 controls. All participants were under 40 years old without cardiovascular disease. Total significant dysrhythmia in 24‐hour ECG monitoring was the primary endpoint determined as the composite of high premature ventricular contraction (PVC) (>500 *beats per* 24 hours), high premature atrial contraction (PAC) (>500 **bp24h**), sinus pause, atrioventricular blocks, ventricular tachycardia, prolonged QTc, and low heart rate variability (HRV). Modified composite dysrhythmia consisted of low HRV (SD of normal‐to‐normal [SDNN]), high PVC and prolonged QT.

**Results:**

Mean heart rate, Efavirenz regimen, male gender, and CD4 count predicted total dysrhythmia. Odds ratios were 1.108, 2.90, 4.36, and 0.96, respectively. The incidence of total dysrhythmia, high PVC, high PAC, low HRV(SDNN), and prolonged QTc were 54.8%, 41.85%, 9.71%, 45.2%, and 12.9% in patients on Efavirenz against 42.11%, 31.64%, 0%, 34.2%, and 7.91% in controls, respectively (*p*‐values: .031, .001, <.0001, .063, and .043 respectively). Modified composite dysrhythmia was also more frequent in Efavirenz group than that of control group (69.42% vs. 52.60%, respectively *p* = .032).

**Conclusions:**

We found that patients with Efavirenz had higher prevalence of frequent PVC, frequent PAC, total significant dysrhythmia, Low HRV and prolonged QTc than controls.

**Abbreviations**
AIDS: acquired immunodeficiency syndrome
ART: anti‐retroviral treatment
AVB: atrioventricular block
bpm: beats per minute
bp24h: beats per 24 hours
CD4: cluster determinant‐4
CRP: C‐reactive protein
ECG: electrocardiogram
HIV: human immunodeficiency virus
HRV: heart rate variability
INSTIs: integrase strand transfer inhibitors
IL‐6: interleukin 6
ms: milliseconds
NNRTIs: non‐nucleoside reverse transcriptase inhibitors
NRTIs: nucleoside reverse transcriptase inhibitors
PAC: premature atrial contraction
PI: protease inhibitors
PVC: premature ventricular contraction
SDANN: standard deviation of the average normal‐to‐normal (NN) 5‐minutes intervals
SDNN: standard deviation of normal‐to‐normal (NN) intervals
VO2 max: volume of O2 consumption
VT: ventricular tachycardia

## 1 INTRODUCTION

Since the start of the HIV‐AIDS outbreak, more than 70 million virus‐infected subjects have been diagnosed and nearly 35 million patients have died. According to the WHO, 36.9 million (31.1–43.9 million) individuals lived with HIV at the end of 2017.^1^ In Iran, the number of infected persons surpasses 60 000 and 37 000 suffer from diseases in the HIV‐AIDS spectrum. The number of patients on appropriate treatment approximates 24 000.^2^ Cardiovascular diseases are now the leading cause of death. Sudden cardiac death accounts for 5%–15% of cardiovascular deaths associated with HIV‐AIDS in the United States, which is assumed approximately 4.5 times higher than expected.^3^ Antiretroviral therapy (ART) have markedly improved survival and quality of life. ART subtends four major categories including non‐nucleoside reverse transcriptase inhibitors (NNRTIs), nucleoside reverse transcriptase inhibitors (NRTIs), integrase strand transfer inhibitors (INSTIs), and protease inhibitors (PIs). An optimal therapeutic protocol usually consists of two NRTIs combined with one drug from the remaining categories.^4^, ^5^ Efavirenz is still one the most commonly used NNRTIs suggested as a first‐line option.^4^ There is a wide variety of cardiovascular potential adverse effects related to ART medications.^6^ However, evidence about increased cardiovascular events or dysrhythmia in patients receiving NNRTIs especially Efavirenz is not enough.^7^, ^8^ Meanwhile we do not have sufficient publications about different types and mechanisms of dysrhythmia in those under Efavirenz treatment. Thus, as a first step we need to evaluate surrogate outcomes such as prolonged QT, which may be precursors of dysrhythmic events. Sudden cardiac death in HIV‐infected patients is linked to previous myocardial infarction, cardiomyopathy, dysrhythmia, and structural heart disease. Of these etiologies, dysrhythmias particularly ventricular tachycardia, may lead to sudden cardiac death in those without evident cardiovascular disease.^3^, ^9^ This study investigates all electrocardiographic features attributable to regimens with and without Efavirenz in patients with no apparent prior cardiovascular disease.

## 2 METHODS

### 2.1 Study participants

This retrospective cohort study was conducted in HIV‐infected patients registered in the electronic database of Iranian Research Center for HIV‐AIDS (IRCHA). Data from 1789 consecutive patients below 40 years of age was extracted according to ID codes. Of these, 23 patients had died before the study started and follow‐up data of 46 patients was missing. Individuals co‐infected with Tuberculosis, hepatitis‐C and/or B were not eligible. Likewise, those with concomitant malignancies, diabetes, hypertension, previous history of cardiovascular diseases including dysrhythmia, family history of sudden cardiac death, and chronic kidney disease were excluded. Current smokers, opiate users, and patients with one or more crossovers of treatment regimens were also excluded. Subjects who stopped treatment for 6 months or longer or did not attend more than four follow‐up visits were excluded. We only enrolled healthy patients (regardless of HIV) who were not under treatment by medications other than ART. Patients were excluded if they had anemia or history of alcohol drinking. Furthermore, none of the participants had elevated blood pressure at baseline records or during visit at the clinic. Ultimately, 134 patients were eligible to participate in the study. Of these subjects, 34 patients either refused to participate in the study or did not have complete baseline data. Thus, a total of 100 patients remained in the study including 62 subjects with and 38 ones without history of receiving Efavirenz.

### 2.2 Study design

A retrospective cohort scheme was accomplished among HIV infected patients aged under the age 40. We sought to investigate the presence of any clinically significant dysrhythmias or their precursor conditions. Hence; all patients who agreed to sign informed consent were visited at cardiology clinic. A comprehensive clinical history regarding cardiovascular disease, medications, hospital admissions, family history of cardiac disorders including sudden arrest was obtained. Thereafter a relevant physical examination accompanied with performing echocardiography were conducted. An ECG Holter monitoring was performed for 24 hours in all participants to evaluate the prevalence of dysrhythmia. All patients were using ART medications while we performed Holter monitoring and the therapeutic regimens continued thereafter. Participants were instructed not to consume caffeine since 24 hours before ECG Holter monitoring. Moreover, they were asked to have their casual daily activities and to avoid moderate or vigorous exercise as well as anxiety. An independent electrophysiologist with collaboration of a cardiologist performed interpretation of 24 hour‐ECG Holter monitoring results. All patients were assessed over a period of 1 month. Baseline laboratory tests such as HIV viral load and CD4 count were collected using the database of our HIV research center. Viral load (as copy numbers) and CD4 counts are markers that help to evaluate the efficacy of treatment and to adjust for the effect of the disease phase. All patients underwent echocardiographic evaluation at baseline and after Holter‐monitoring in order to detect any considerable structural heart disease.

### 2.3 Definitions

Primary endpoint of the study was total significant dysrhythmia. It was defined as a composite of frequent premature ventricular contraction (PVC) (>500 **bp24h**), high premature atrial contraction (PAC) burden (>500 **bp24h**), sinus arrest, atrioventricular blocks, ventricular and supraventricular tachycardia, prolonged QTc, and low heart rate variability (HRV) (low SD of the average normal‐to‐normal [SDANN]). In addition, we defined another modified composite endpoint which consisted of low SD of normal‐to‐normal (SDNN), prolonged QT, and frequent PVC. These components seem to have greater clinical relevance with Efavirenz. High burden of ectopic beats (>500 **bp24h**) was determined according to prior studies.^10^ We. Decreased adjusted HRV was determined as SDNN under 50 ms or SDANN values below the fifth percentile.^11^ SDNN represents SD of NN intervals whereas SDANN refers to the standard deviation of the averages of NN intervals in all 5 minutes segments of the ECG.^12^ QT intervals were calculated using the baseline ECG. Corrected QT intervals were calculated via the Bazett's formula.^13^ Various sex‐based cutoff points have been suggested to discriminate patients with prolonged QT based on different calculations. However, there is a general agreement about values of the Bazett's formula as QT > 450 ms, and QT > 460 ms in males and females, respectively.^14^ Glomerular filtration rate (GFR) was calculated with the modification of diet in renal disease formula.^15^

### 2.4 Statistical analysis

Continuous variables are expressed as mean ± SD while categorical variables are presented as number and/or percentages. Two‐tailed student's *t*‐test and Mann–Whitney *U* test were used to compare continuous variables with and without normal distribution, respectively. *χ* ^2^ test or Fisher's Exact Test were used to detect differences in categorical variables. Multivariate logistic regression was applied to define independent predictors of total significant dysrhythmia. *p* values less than .05 were considered significant. We performed the data analysis using IBM SPSS Statistics for Windows, version 23.0 (Armonk, NY: IBM Corp).

## 3 RESULTS

The mean age of the patients was 34.22 ± 5.11 years and the median treatment length (follow‐up) was 6.31 years. Sixty‐two participants were on treatment with Efavirenz‐containing regimens and 38 subjects received non‐ Efavirenz regimens. Table 1 presents the baseline general characteristics of the two groups. The majority of the patients (98%) used NRTI drugs whereas only 6% were on INSTIs. About 68% of the participants were treated with PIs. Baseline left ventricular ejection fractions (LVEF) of the subjects with and without Efavirenz were similar (median values were 52.5% and 55% respectively, *p* = .64). Patients in this study were asymptomatic. None of them had experienced chest pain, exertional dyspnea; peripheral edema, palpitations, orthopnoea, and syncope. All of them underwent Echocardiography, which did not reveal structural heart disease including regional wall motion abnormalities. Follow‐up echocardiography revealed preserved LVEF and normal right ventricle function in all participants. Ejection fractions were identical on follow‐up (median values were 55 for both groups with a *p*‐value of .85). Table 2 shows the significance of different predictors of the composite endpoint, that is, total significant dysrhythmia, regarding multiple adjustments, which resulted in two models. Table 3 presents the cumulative prevalence of primary and secondary endpoints according to subgroups of gender and treatment duration. There were two cases with uncommon dysrhythmias, which were found incidentally. One had asymptomatic intermittent manifestation of a delta wave. The other patient had prominent alterations in the amplitude and direction of T waves known as T‐wave alternans. No episodes of sustained or non‐sustained VT occurred, not even in the subgroup of patients with prolonged QT intervals. Prolonged QT interval was more frequent in patients who did not use PIs although the difference was not significant (10% vs. 6.2%, *p* = .24). Treatment with PIs tended to be associated with fewer events of total dysrhythmia (54% vs. 42%, *p* = .065).

**TABLE 1:** Baseline characteristics of the study participants with respect to Efavirenz regimen

	Non‐Efavirenz (*n* = 38)		Efavirenz (*n* = 62)		*p*‐value
Age	34.47 ± 5.38 (32.71–36.24)		35.31 ± 4.51 (34.16–36.45)		.54
Sex (male)	60.51% (23)		66.1% (41)		.082
NRTI	97.36% (37)		98.38%(61)		. 68
INSTI	5.26%(2)		6.45%(4)		.152
PI	76.31%(29)		62.90%(39)		.012
BMI (kg/m^2^)	30.47 ± 3.76 (26.71–42.68)		32.47 ± 5.16 (23.71–45.24)		.85
*Follow‐up time 1 (duration of active treatment)	*6.25 (2–9)		*4.5 (1.5–7)		.19
*Disease duration (duration since diagnosis of HIV infection)	*7.75 (2.5–9.5)		*6.5 (2–8)		.31
CD4 count	600.78 ± 73.81 (519.21–682.36)		570.61 ± 123.51 (497.93–641.25)		.58
*Final viral load	1587.60 ± 93.70 (543.30–10618.51)	2134.5(450)*	1036.60 ± 58.30 (447.30–8629.81)	1806.5(629.5)*	.47
GFR (ml/min/1.73 m^2^)	108.11 ± 12.34 (76.65–136.18)		102.76 ± 18.09 (68.05–143.53)		.78
Baseline QTc	435.12 ± 22.04 (385.65–511.14)		428.02 ± 26.84 (378.60–501.64)		.58
Mean heart rate	81.42 ± 9.52 (78.29–84.55)		81.38 ± 7.194 (79.59–83.25)		.41

*Note*: Continuous variables are expressed as Mean ± SD (95% CI) or Median (Interquartile range) if * is assigned to the variable.

Abbreviations: BMI, body mass index; CD4, cluster determinant 4; GFR, glomerular filtration rate; INSTIs, integrase strand transfer inhibitor; NRTI, nucleoside reverse transcriptase inhibitor; PI, protease inhibitor.

**TABLE 2:** Multivariate logistic regression to evaluate the association of adjusted effects of independent predictors of Total significant arrhythmia

Predictors of composite endpoint	Odds ratio 1	95% CI for OR	*p*‐value	Odds ratio 2	95% CI for OR	*p*‐value
Age (per 5 years increase)	1.09	(0.961–1.247)	.227	1.074	(0.959–1.204)	.216
Longevity of treatment (>5 vs. <5 years)	0.92	(0.231–1.83)	.371	1.087	(0.951–1.242)	.222
Mean HR (per 10 bpm increase)	1.187	(1.075–1.312)	.001	1.108	(1.033–1.190)	.004
Efavirenz	2.46	(1.23–6.084)	.031	2.90	(1.062–7.921)	.038
Sex (male vs. female)	4.357	(1.34–14.13)	.014	4.357	(1.234–8.469)	.017
Protease inhibitor	0.422	(0.039–4.56)	.477			
CD4 count (per 100 units increase)	0.96	(0.88–1.601)	.058			

*Note*: Odds ratio 1 shows values after multiple adjustments for other ART medications (INSTI and NRTI), body mass index, hemoglobin level, baseline LV ejection fraction, and final viral load of HIV. Odds ratio 2 represents the likelihoods with adjustments mentioned above in addition to considering baseline QT interval in 12 lead ECG, and renal function (GFR).

**TABLE 3:** Clinical and electrocardiographic outcomes with respect to Efavirenz consumption stratified by different subgroups

	Non‐Efavirenz (%)	Efavirenz (%)	*p*‐value
Total significant dysrhythmias	42.11	54.8	.031
Male	6.67	51.22	<.0001
Female	65.22	61.90	.82
TD <5 years	35.74	44.19	.047
TD >5 years	45.83	67.86	.035
Frequent PVC	31.64	41.85	.001
Male	13.30	43.90	<.0001
Female	43.48	38.10	.37
TD <5 years	14.3	35.38	.025
TD >5 years	41.7	50.03	.091
Frequent PAC	0.0	9.71	<.0001
Male	0.0	14.61	<.0001
Female	0.0	0.0	NA
TD <5 years	0.0	14.7	<.0001
TD >5 years	0.0	3.60	.350
Low HRV* (SDANN<5th percentile)	23.72	24.26	.28
Male	0.0	19.51	<.0001
Female	39.13	33.33	.14
TD <5 years	7.13	14.71	.16
TD >5 years	33.34	35.70	.86
Low HRV* (SDNN<50)	34.2	45.2	.063
Male	13.3	48.8	<.0001
Female	43.4	42.8	.515
TD <5 years	35.7	41.2	.15
TD >5 years	33.3	50.0	.016
Prolonged QT	7.91	12.90	.043
Male	0.0	9.80	.012
Female	13.04	19.05	.335
Tx <5 years	14.29	2.94	.143
Tx >5 years	4.2	25.0	<.0001
AV block (second/third degree)	8.70	11.96	.41
Male	0.0	2.44	.54
Female	8.70	9.52	.93
VT	0.0	0.0	NA
Sinus pause/sinus arrest	5.33	6.54	.78

Abbreviations: AV, atrioventricular; HRV*, heart rate variability; NA, not applied; PVC, premature ventricular contraction; SDANN, SD of the average normal‐to‐normal (NN) 5‐minutes intervals; SDNN, SD of normal‐to‐normal (NN) intervals; TD, Treatment duration; VT, ventricular tachycardia.

**FIGURE 1:**
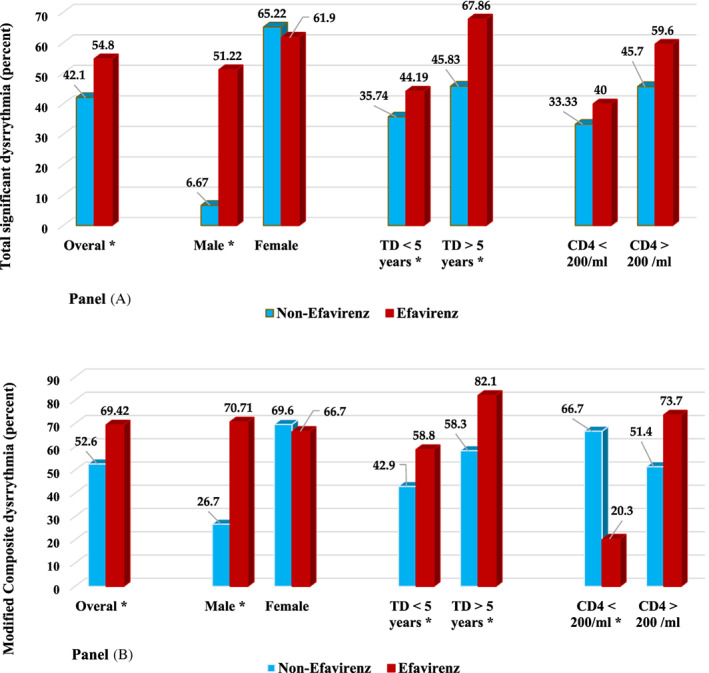
Frequencies of composite endpoints based on comparison between Efavirenz and non‐Efavirenz regimens with further subgroup analysis. (A) Depicts the prevalence of Total significant dysrhythmia. (B) Represents distribution of the Modified composite endpoint, which consists of three components including low HRV (SDNN), frequent PVC, and prolonged QTc. TD refers to treatment duration. CD4, cluster determinant 4. A subgroup with *p*‐value <.05 is marked via *

Table 4 presents univariate and multivariate analysis pertaining to the predictors of modified composite endpoint. Furthermore, Figure 1 illustrates the differences between the two composite endpoints according to observed frequencies in subgroups.

**TABLE 4:** Multivariate logistic regression to evaluate the predictors of Modified composite dysrhythmia

Predictors of composite endpoint	OR 1 (univariate)	95% CI	*p*‐value	OR 2 (multivariate)	95% CI	*p*‐value
Age (per 5 years increase)	1.14	(0.83–1.54)	.430	1.05	(0.94–1.18)	.172
Longevity of treatment (>5 vs. <5 years)	1.71	(1.02–2.33)	.021	1.214	(1.04–1.41)	.012
Mean HR (per 10 bpm increase)	1.32	(1.11–1.85)	.005	1.105	(1.027–1.18)	.007
Efavirenz	2.76	(1.20–8.15)	.028	2.13	(1.14–6.02)	.011
Sex (male vs. female)	3.65	(1.04–9.18)	.037	1.750	(0.65–4.72)	.274
Viral load	1.82	(0.76–5.41)	.292	1.09	(0.86–2.65)	.216
CD4 count (per 100 units increase)	0.91	(0.73–1.72)	.081	0.95	(0.55–1.05)	.054

*Note*: Odds ratio 1 shows univariate logistic regression. Odds ratio 2 considers multiple adjustments for ART drugs (INSTI, PI, and NRTI), body mass index, baseline LV ejection fraction, baseline QT interval in 12 lead ECG, final viral load, and renal function (GFR).

## 4 DISCUSSION

We focused on QT prolongation, PVC, PAC, brady‐arrhythmia, and decreased HRV which mediate development of potentially malignant dysrhythmia such as VT. We found that patients on Efavirenz had increased prevalence of high PVC, high PAC, and total significant dysrhythmia than those not treated with Efavirenz. Similar rates of low HRV were detected in both groups. Prolonged QTc were observed in both groups. There is controversy about the impact of Efavirenz on QT length regardless of its subsequent clinical manifestations.^9^, ^16^, ^17^, ^18^, ^19^ Nevertheless, most studies such as the one by Charbit et al., found that prolonged QT is associated with conventional factors as well as chronicity of HIV infection rather than with ART drugs.^20^ We found that Efavirenz regimen increased the length of corrected QT interval overall and in males while females were not affected. Indeed, male subjects on Efavirenz were more likely to have prolonged QT (9.80% vs. 0%) as compared to non‐Efavirenz men. Impact of Efavirenz on QT is not well established. Few studies have noticed about that Efavirenz might prolong QT interval, which is in line with our results.^21^ Conversely, prolonged QTc was more frequent in females than in males irrespective of Efavirenz use. Different regulation of potassium channels called Ikr (rapidly activating delayed rectifier) is well established in women compared with men. This is concordant with many reports such as the study by Hreiche and co‐workers.^22^

The pathophysiology underlying QT prolongation by ART drugs is not well understood. However, decreased outward potassium, currents over the second phase of action potential has been proposed. Low expression of the human Ether‐a'‐go‐go related gene (HERG) might control these potassium‐rectifier channels which leads to extended repolarization.^23^ Then, prolonged QTc may serve as a preliminary state to trigger premature ventricular beats (PVB) as well as ventricular tachyarrhythmia (VT).^24^ Regarding vulnerable genotypes, studies have shown that risk of Efavirenz ‐mediated long QT markedly increased in homozygous carriers of the CYP2B6*6 allele.^9^, ^18^ In addition to ART regimens, polypharmacy might cause interactions and provoke dysrhythmias. With this in mind, we excluded patients who currently used medications other than ART, illicit drugs or smoked tobacco. Overall, the multi factorial nature of QT prolongation and various confounding factors in HIV‐AIDS is a challenge but caution should be kept when prescribing specific ART components such as Efavirenz.

Prolonged QT might also appear secondary to autonomic dysregulation, which is associated with both virus‐mediated and ART‐induced alterations in sympatho‐vagal balance. Although diminished autonomic reserve usually occurs in the early stages of HIV infection, severe dysfunction emerges in late phase of the disease. However, the duration of disease and the longevity of treatment regimens were both similar in Efavirenz group compared with non‐Efavirenz patients. Previous studies have reported considerable decline in HRV of HIV patients on ART regimens, particularly NNRTIs like Efavirenz.^25^ SDANN is a measure of total HRV indicating the effects of both sympathetic and parasympathetic derives. General metabolic disturbance either due to HIV infection or to medications may contribute to low parasympathetic activity and low SDANN.^26^ Moreover, lipodystrophy occurs commonly in patients receiving Efavirenz.^27^ Some researchers declared that hypercholesterolemia, and hyperglycaemia diminish the HRV regardless of the duration of ART, which is discordant with our findings.^28^ In fact, we observed greater prevalence of low HRV (SDNN) and prolonged QT in Efavirenz patients (against non‐Efavirenz group) who were treated for more than 5 years. A meta‐analysis by Mc Intosh RC found that HRV was lower in a cohort of HIV‐infected young adults who received ART than those without ART.^29^

HRV serves as a marker of cardiopulmonary fitness. Multiple studies have focused on exercise‐based interventions in HIV‐infected individuals. Grant and colleagues examined the influence of a regular aerobic exercise protocol on SDANN and other measures of HRV. Their controlled trial found improvement of maximal volume of oxygen uptake (VO2max) and incremental values of HRV after 16 weeks.^30^ Recent studies have shown an inverse relationship between biomarkers of inflammation such as IL‐6, CRP, and tumor necrosis factor, and HRV.^31^ Similar associations have been shown for the coagulation cascade factors like D‐dimer in HIV patients.^32^, ^33^

In the present study, Efavirenz affected male subjects more than females. In fact, total significant dysrhythmia and most of the components except AV block and sinus pause were more frequent in males on Efavirenz than in males not on Efavirenz. Nevertheless, females on Efavirenz did not differ from females of non‐Efavirenz group with regard to any type of dysrhythmia. Given these gender differences, it seems that higher levels of inflammatory cytokines accompanied by electrical remodeling in patients with active HIV infection rather than those with controlled status^9^ Potential alterations in ion channels and autonomic drive in HIV infected males under ART were indicated in few studies.^18^, ^34^ Thus, precursors of dysrhythmia like reduced HRV and prolonged QT were associated with prominent inflammation in HIV infected men.^34^, ^35^ Our findings of long QT are in line with these reports. However, subgroup analysis demonstrated longer QTc in males under Efavirenz as compared with males in control group. Furthermore, there was a high rate of prolonged QTc in patients with longer treatment courses (>5 vs. <5 years).

A greater PAC burden (>500 bp24h) was found in male HIV‐AIDS patients who received Efavirenz‐containing regimens. This finding was not mentioned in any of the prior studies, which evaluated ART‐associated dysrhythmia. We did not detect any cases with complete AV block, but second‐degree blocks were similar in the two groups. Therefore, as described in prior studies, Efavirenz may not contribute to an increased rate of blocks and other conduction disturbances in HIV‐infected patients.^36^, ^37^, ^38^

Despite general similarity of the two groups at baseline, a higher proportion of non‐Efavirenz patients received PI. Treatment with PIs was not associated with dysrhythmia or significant ECG changes. This is in accordance with studies by Charbit et al.^20^ and Fiorentini et al.^17^ Despite case reports and initial studies^39^ supporting such an effect, cumulative evidence has shown that QTc prolongation is not directly related to PIs.^17^, ^20^, ^40^, ^41^

Multivariate model in our study showed a trend toward significant decrease of dysrhythmia with greater CD4 counts which was close to previous reports.^42^ This may underline the role of optimal treatment for reducing dysrhythmias particularly prolonged QT.

We excluded patients with a history of diabetes, hypertension, coronary artery disease, current smoking, dysrhythmia, chronic kidney disease, and family history of sudden cardiac death, since these factors might increase susceptibility to ischemic heart disease, which could lead to dysrhythmias. Therefore, included patients constituted a relatively homogeneous population of young to middle‐aged HIV patients who were apparently free of cardiovascular disease. Although the pathogenesis of dysrhythmia involves multiple pathways and aetiologies, we aimed to restrict underlying risk factors, particularly overt structural heart diseases. Since potential confounding factors were eliminated, we could demonstrate effects attributable to antiretroviral regimens.

## 5 STUDY LIMITATIONS

The present investigation was a single‐center study. However, it was conducted in a tertiary referral hospital with specialized facilities for HIV patients. The retrospective design poses some caveats pertaining to causal and temporal linkages between exposure and outcomes. Methodological barriers prevented us to evaluate if Efavirenz, and other ART drugs, are risk factors or independently causative. The total number of malignant dysrhythmias such as VT was low which may mask the true impact of the Efavirenz. Therefore, further studies are required to evaluate all types of rhythm disorders secondary to Efavirenz. Ventricular and atrial ectopic dysrhythmias usually vary with time, which mandates monitoring for longer periods such as 48 hours or even 1 week.

As discussed earlier, we did not measure levels of inflammatory biomarkers to evaluate their impact. Clinical implication of the observed outcomes, which are precursors of dysrhythmia remains to be elucidated in larger studies with, extended follow‐up times. Cardiovascular events such as syncope, sudden arrest, and stroke should be targeted. Furthermore, whether continuing Efavirenz or substituting with another drug prevents cardiac events remains uncertain. Our findings might be restricted to a particular subgroup of otherwise healthy HIV patients, which limits generalizability. Additionally, the method for detection of dysrhythmias has limitations for relatively rare or infrequent episodes of certain disorders.

## 6 CONCLUSION

Patients exposed to Efavirenz had higher prevalence of high PVC, high PAC, and total significant dysrhythmia but similar proportions of low HRV (SDANN). However, Efavirenz group had lower HRV as measured via SDNN with a borderline significance. Efavirenz regimen, mean heart rate, male gender and lower CD4 counts were independent predictors of total dysrhythmia. Efavirenz regimen increased QT, which was prominent in subgroup of males while females were not affected. Furthermore, majority of impacts exerted via Efavirenz were predominantly observed in males rather than females. Use of ambulatory electrocardiographic monitoring may improve screening and diagnosis of at risk individuals. However, the subgroup of HIV‐infected patients who may benefit most from screening of dysrhythmia, the appropriate time intervals, methods, cost‐effectiveness, follow‐up protocols, and management algorithms are unclear.

## CONFLICT OF INTEREST

The authors declare that they had no type of competing interests.

## AUTHOR CONTRIBUTIONS

Reza Mollazadeh and Seyed‐Ali Dehghan‐Manshadi provided the core concept of the case report and performed treatment of the patient as well as supervising the study. Zahra Hosseini and Ali Akbari collected the data. Zahra Hosseini, Mehrnaz Mohebi, and Saeed Ghodsi wrote the draft and revised the manuscript. Zahra Hosseini, Seyed‐Ali Sadre Bafghi, Seyed‐Ali Dehghan‐Manshadi, and Mehrnaz Mohebi prepared the tables and Figures. Saeed Ghodsi and Zahra Hosseini performed statistical analysis. All authors read and approved the final manuscript.

## INFORMED CONSENT

We obtained written informed consent from all patients for publishing the clinical data and related aspects.

## Data Availability

Data sharing will be applicable to this article according to probable requests in the context of a formal agreement.
